# Factors influencing health-related quality of life of children with overweight and obesity in Kuala Lumpur, Malaysia

**DOI:** 10.1080/21642850.2024.2413980

**Published:** 2024-10-16

**Authors:** Shazana Rifham Abdullah, Hoe Victor Chee Wai, Zahari Ishak, Siti Sarah Hamzah, Ahmad Kamil Nur Zati Iwani, Ruziana Mona Wan Mohd Zin, Abqariyah Yahya

**Affiliations:** a Nutrition, Metabolism and Cardiovascular Research Centre, Institute for Medical Research, National Institute of Health, Ministry of Health, Selangor, Malaysia; b Department of Social and Preventive Medicine, Faculty of Medicine, Universiti Malaya, Kuala Lumpur, Malaysia; c Department of Educational Psychology and Counseling, Faculty of Education, Universiti Malaya, Kuala Lumpur, Malaysia

**Keywords:** Health-related quality of life, overweight, obesity, children, adolescents

## Abstract

**Introduction:**

Overweight and obesity among children may have psychological consequences, with potentially lasting effects on health-related quality of life (HRQOL). The aims of this study were to compare HRQOL across weight status among children and to determine the factors influencing HRQOL among children with overweight and obesity.

**Methods:**

This cross-sectional study involved school children aged 9–16 years that were recruited from eight randomly selected primary and secondary schools in Kuala Lumpur. The validated Malay version of Pediatric Quality of Life Inventory (PedsQL) was used to measure HRQOL. Complex sample general linear model was used to determine the significant factors associated with HRQOL among children with overweight and obesity.

**Results:**

From 928 students, 41.2% (n* *= 375) of them had either overweight or obesity. Children with obesity reported lower overall HRQOL, physical functioning, social functioning, and psychosocial health summary, compared to normal weight children. In the final model, children with obesity had significantly lower HRQOL and physical functioning compared to children who were overweight, while those who lived with a single parent reported poorer HRQOL compared to children who lived with both parents. Children with history of being bullied had lower HRQOL and psychosocial health. Whereas those with lower self-esteem reported significantly lower scores in all three domains.

**Conclusion:**

Overweight and obesity have negative impacts on HRQOL of children. Among children with overweight and obesity, factors such as degree of obesity, family structure, history of being bullied, and self-esteem were found to be significantly associated with HRQOL. Therefore, assessing and managing HRQOL should be included as a part of the obesity prevention programme.

## Introduction

Obesity is an escalating global epidemic, affecting practically all countries regardless of socio-economic status as well as age groups (World Health Organization, 2022). Globally, more than 360 million children aged 5–19 years were with overweight and obesity in 2016. In 2019, nearly half of the children under five who had either overweight or obesity lived in Asia. Although childhood obesity was once considered a high-income country problem, it is now on the rise in low – and middle-income countries, particularly in urban areas (World Health Organization, 2020). In Malaysia, the National Health and Morbidity Survey reported in the Adolescent Health Survey 2022 that approximately 30.5% of Malaysian adolescents had either overweight or obesity, with females being more prevalent in the overweight category (16.5%) (Institute for Public Health, 2022).

Obesity among children is associated with numerous health consequences, not only limited to their physical health but also their psychosocial health (Williams et al., 2005). Health-related quality of life (HRQOL) is defined as an individual’s quality of life (QOL) associated with their physical, mental, and social well-being (World Health Organization, 2001). Studies showed that children with overweight and obesity have an overall impaired HRQOL compared to their normal-weight peers (Abdel-Aziz et al., 2014; Al-Akour et al., 2012; Anderson et al., 2017; Brodsgaard et al., 2016; Lee et al., 2018; Lin et al., 2013; Meixner et al., 2020; Wong et al., 2019).

For children with overweight and obesity, excess weight can be socially and emotionally challenging (Lin et al., 2018). With a mixture of extra awareness towards body image, body dissatisfaction, social discrimination, and social stigma (Chan et al., 2019), obesity has the power to affect children’s QOL. Children with overweight and obesity are often teased or bullied for their weight and image, besides encountering numerous other oppressions, including discrimination, and social marginalisation (Bacchini et al., 2015), and often suffered poor quality of life (Haraldstad et al., 2011; Radhakishun et al., 2016), negative emotional consequences (Lee et al., 2018), academic failure (Puhl & Luedicke, 2012) and peer rejection (Lumeng et al., 2010).

According to the Socio-Ecological Model (SEM), there are multifaceted and interactive effects of personal and environmental factors that determine behaviours (Kilanowski, 2017). It can provide a useful framework to better understand the many factors and barriers that impact health behaviours and outcomes, including HRQOL (Townsend & Foster, 2013). Literature shows that individual factors such as sex, age, ethnicity, BMI z-score, and self-esteem have been significantly associated with poor HRQOL (Chen et al., 2015; Haraldstad et al., 2011; Kolodziejczyk et al., 2015; Meixner et al., 2020), as well as interpersonal and community factors such as parental educational status, family structure, household income, and bullying (Al-Akour et al., 2012; Radhakishun et al., 2016; Rattay et al., 2018; Simon et al., 2008).

### Purpose of study

To date, there are limited studies that have explored HRQOL among children with overweight and obesity in Malaysia. For instance, in a study by Lee et al*.* (Lee et al., 2012), HRQOL was compared between children with obesity and those of normal weight. However, similar to Hamzaid et al. (Hamzaid et al., 2011), they did not further investigate what factors significantly influenced the HRQOL. Thus, our study attempted to address this gap by examining factors associated with HRQOL among children with overweight and obesity in the Malaysian context, since there are different dynamics in the social-emotional development of children even among Asian countries (Yong et al., 2023). Furthermore, determining the factors associated with HRQOL among this population will provide more valuable evidence and new insight for a better approach to planning preventive measures. The specific research questions of this study were: (1) Is there any difference in HRQOL between children who had normal weight, overweight, and obesity?, and (2) What are the factors associated with HRQOL among children with overweight and obesity? Thus, this study aimed to compare HRQOL across weight status among children and determine whether age, sex, ethnicity, school category, family structure, presence of asthma, parents’ educational status, household income, history of being bullied, degree of overweight, or self-esteem are the associated factors of HRQOL among children with overweight and obesity in Kuala Lumpur, Malaysia.

## Methods

### Participants

This cross-sectional study was conducted among schoolchildren aged 9–16 years old between March and November 2017. We collected data from both primary and secondary schools in Kuala Lumpur. Students from standards three, four, and five in primary school (aged 9–11 years) and forms one, two, and four in secondary school (aged 13, 14, and 16 years) were included in this study. Children aged 12, 15, and 17 years were excluded, as students in major exam years were not permitted to participate in school-based research. Those who were diagnosed with any physical or mental disabilities were excluded. Those who were not able to read in the Malay language were also excluded. Written informed consent was obtained from either parents or guardians, and assent was obtained from the children. This study was approved by the University of Malaya Medical Centre Ethics Committee (Grant Number: 20162–2149).

The sample size was calculated using Power and Sample Size software (Dupont & Plummer, 1990). The sample size was calculated using the mean difference derived from a study by Lee et al. (Lee et al., 2012). With a confidence level of 95% and a power of 80%, the minimum sample size required was 358. By considering 40% of the non-response rate and a design effect of 1.3, the final sample size needed was 626 subjects, with 313 of them were with overweight or obesity. The design effect used to calculate the sample size was derived from our pilot study.

Cluster sampling was conducted using methods that ensured that representative samples of children in Kuala Lumpur were recruited. A list of primary and secondary schools in Kuala Lumpur was obtained from the Kuala Lumpur Education Department. The total number of primary schools was 141, while secondary schools were 90. The schools were randomly selected using the Statistical Package for the Social Sciences (SPSS) version 21 software (IBM Corp, 2012). Based on the estimation that there are 50 students with overweight and obesity in each school, a total of 12 schools were needed to meet the sample size. From the list of schools, six primary schools and six secondary schools were randomly selected. However, the minimum sample size was achieved from only eight schools, that was, four primary and four secondary schools.

### Measures

a) Pediatric Quality of Life Inventory (PedsQL)

The Malay version of the Pediatric Quality of Life Inventory (PedsQL) was used to measure HRQOL (Varni et al., 2001). Varatharajan and Chen (Varatharajan & Chen, 2013) have validated the Malay version of the PedsQL questionnaire. The Cronbach's alpha values in Malay versions of PedsQL ranged between 0.80 and 0.92, which indicates the instrument has good internal validity and reliability (Tavakol & Dennick, 2011). A moderate correlation was observed for children aged 5–7 and 8–12 years, while the correlation was moderate to high for children aged 13–18 years. Permission to use the PedsQL 4.0 Generic Core Scales was obtained from the original author (Varni et al., 2001). A user agreement was signed between the Mapi Research Institute, Lyon, France, and the researcher.

Two versions of the scale were used according to the ages of the children, namely, children aged 8–12 years and adolescents aged 13–18 years. The scale contained 23 items, which were divided into four domains: physical functioning (eight items), emotional functioning (five items), social functioning (five items), and school functioning (five items). The psychosocial health summary score was the average score of three domains: emotional, social, and school functioning. The scale comprised a five-point Likert scale (0 = never a problem, 1 = almost never, 2 = sometimes, 3 = often, and 4 = almost always). To obtain the total score, each item was transformed to a 0–100 scale (0 = 100, 1 = 75, 2 = 50, 3 = 25, 4 = 0). Scale scores were computed as the sum score of the items divided by the number of items answered, and higher scores indicate better HRQOL (Varni et al., 2001).

In this questionnaire, the children were asked how much each item had affected them during the past month. The physical functioning domain consists of questions concerning physical health and activities, such as how much difficulty they had walking more than one block, doing chores around the house, and doing sports activities. The emotional functioning domain consists of questions pertaining to their emotional feelings, while assessments on whether they had problems getting along with other kids were asked in the social functioning domain. In the school functioning domain, questions about forgetfulness, difficulty paying attention in class, and problems in keeping up with their schoolwork were asked.

b) Socio-demographic factors

The factors examined in this study include age (Haraldstad et al., 2011), sex (Chen et al., 2015; Meixner et al., 2020), ethnicity (Radhakishun et al., 2016), school category, family structure (Simon et al., 2008), presence of asthma, parents’ educational status (Chen et al., 2015), household income (Simon et al., 2008), history of being bullied (Haraldstad et al., 2011; Radhakishun et al., 2016), degree of overweight (Chen et al., 2015; Kolodziejczyk et al., 2015), and self-esteem score (Haraldstad et al., 2011). The selection of these variables was based on literature reviews that identified their significant link with HRQOL.

Information on children's age, ethnicity, gender, parents’ educational status, household income, asthma, history of being bullied, and family structure were obtained from the socio-demographic part of the questionnaire. The highest parental educational status was obtained for both mother and father. The choices of answers were: (1) non-formal education, (2) primary school, (3) SRP/PMR (lower secondary assessment), (4) SPM/STPM (Malaysian Certificate of Education/Malaysian Higher School Certificate), (5) diploma, (6) degree/master/PHD, and (7) others. The options were then further categorised into three categories: (1) no formal education, (2) low education status (primary school to SPM/STPM), and (3) high education status (diploma to degree/master/PhD).

For household income, the information was obtained from both parents separately. It was assessed in continuous data and was categorised based on income class for the lower 40% (less than RM2300), the middle 40% (between RM2300 to RM5599), and the high 20% (≥ RM5600) according to the Tenth Malaysia Plan (RMK10) classification (Zainuddin et al., 20).

In the family structure, a two-parent family was defined as a family with a biological mother and father staying in one household, while a parent could be deceased or living separately due to divorce in a single-parent family. The information was obtained through the question, ‘What is your parents’ marital status?’. The options were (1) married and (2) single parent.

Bullying was measured by asking, ‘Have you experienced being bullied by other children or adolescents?’ The options of response were ‘Yes = 1’ or ‘No = 0’. There were no further questions on the source or type of bullying.

The classification of overweight and obesity in this study was based on the WHO definitions. Overweight and obesity were defined as BMI z-scores of more than +1 and +2 standard deviation, respectively, while normal was defined as BMI z-scores of between – 2 and +1 standard deviation, and underweight was less than – 2 standard deviation (thinness < −2 s.d.; severe thinness <3 s.d.), respectively, for their age and sex (de Onis et al., 2007).

c) Self-esteem

Self-esteem was measured using the Malay version of the Rosenberg Self-Esteem Scale (RSES). It is a 10-item scale that measures global self-worth by considering both positive and negative feelings about oneself. The positive feelings that were assessed on this scale include self-satisfaction, self-respect, and self-ability. In examining the negative feelings, the children were asked whether they had experienced any feelings of uselessness, had nothing much to be proud of, and considered themselves a failure. All items were answered using a 5-point Likert scale, ranging from strongly agree to strongly disagree. Questions 1, 2, 4, 7, 8, and 10 were positively worded items, while questions 3, 5, 6, and 9 were negatively worded items and were reversely coded in the calculation of the total score. The scores were summed: higher scores indicate higher self-esteem (Yaakob, 2015). The Malay version of the RSES questionnaire had been validated by Yaakob (Yaakob, 2015) among schoolchildren in Seremban with a Cronbach’s alpha value of 0.8. Overall, the Malay version of RSES is a valid and reliable tool for assessing self-esteem.

### Procedures

Prior to data collection, meetings with school representatives were conducted in which the researcher explained the study's aims, procedures, and ethics. Once permission was granted, a list of students (names) who fulfilled the inclusion criteria was obtained. Arrangements were then made for two sessions of data collection based on the schedule and convenience of the school.

During the first session of data collection, students were briefed on the study's purpose and procedures. Each student then received a set of documents, which consisted of a formal invitation letter, brochure, participant information sheet, consent forms, and a questionnaire to bring home and show their parents. Once consented to, the students and parents then completed the questionnaire provided. They were free to contact and consult the researcher at any time if they had any difficulty understanding the questionnaire or had any enquires regarding the study.

The second session of data collection was carried out a day later. Questionnaires and consent forms were collected prior to the measurement of anthropometric data (height, body weight, and body fat percentage). All enquiries regarding the questionnaire were answered during this session. Weight was measured with light clothing without shoes to the nearest 0.1 kg using a pre-calibrated body composition monitor (Omron Karada Scan, China). Height was measured without shoes to the nearest 0.1 cm using a portable standardised stadiometer.

### Analysis plan

The data was analysed using the SPSS version 25 software (IBM Corp, 2012). Given the cluster random sampling method employed in this study, complex sample data analysis adjusted for the weight of the sampling design was performed. The complex sample analysis plan file was prepared prior to analysis. The total weightage (FWST.SC) was calculated by multiplying the students’ and schools’ weightage. The school’s weightage (WSC) was derived by dividing the total number of schools by the number of selected schools within the sampling frame. The student’s weightage (WST) was obtained by dividing the total number of students from each school by the number of participants from the same school.

A complex sample analysis was then conducted using the general linear model for all analyses. The data was checked against the assumptions of the data analysis methods. Socio-demographic data were described according to BMI z-score classes. Continuous data was presented as mean and standard error (SE). For categorical data, frequencies were calculated and presented with percentages. The HRQOL scores, including total and all domains, were then compared between weight status (normal weight, overweight, and obese), controlling for age, sex, ethnicity, and household income. Students who were underweight were excluded from the analyses as the topic is beyond the scope of this study.

To determine the factors associated with HRQOL, a sub-analysis was conducted among those with overweight and obesity. The independent association between HRQOL and gender (Meixner et al., 2020), age, ethnicity, type of school, degree of overweight, history of being bullied, parents’ education status, household income, family structure, presence of asthma, and self-esteem score was first examined using complex samples univariate analyses (results not presented). Variables that were clinically important or with a *p*-value <0.25 or both from the univariate analyses were then included in the model to determine the significant predictors for total PedsQL score, physical functioning, and psychosocial health summary among children with overweight and obesity. Due to its clinical importance, gender was included in all three multivariate models, although the *p*-value of independent association in the univariate analyses was <0.25 (Al-Akour et al., 2012; Meixner et al., 2020). Similarly, household income (Al-Akour et al., 2012) and age (Haraldstad et al., 2011) were included in the Total PedsQL and Physical Functioning models, respectively. All analyses were conducted 2-sided and significant level was set at 0.05.

## Results

### Sample description

Of the 2674 students who were recruited, only 928 participated. The main reasons for exclusion were no parental consent and students failing to present the consent forms to their parent or guardian. More than half of the participants had normal weight (n = 488, 52.3%), followed by overweight (n = 193, 21.3%) and obesity (n = 182, 19.9%), with only 6.5% (n = 59) in the underweight category. There was a significant difference in the distribution of sex across weight status, as there was a nearly equal distribution of boys and girls among children (48.2% versus 51.8%), compared to the other groups, which were predominated by girls (*p *< 0.001). Most children with obesity were from primary school (n = 111, 64.1%), which significantly differed from the distribution of children based on the school category in other weight statuses (*p *< 0.004). There were 15.6% (n = 143) of children that had a history of being bullied, with a proportion within the overweight group being significantly lower (9.3%) compared to other weight status groups, especially the underweight and obese groups (both 21.3%). Children with obesity were significantly younger (mean = 11.7, SE = 11.3, *p *= 0.031) than children in other weight status groups. In view of parents’ educational status, most parents reported receiving education up to a lower educational level [mother (n = 607, 66.3%), father (n = 585, 66.4%)]. For household income per month, 37.9% (n = 335) of children were from middle-income families, followed by high-income families (n = 297, 33.7%) and low-income families (n = 245, 28.4%) (Table 1).

**Table 1.** Characteristics of Children by BMI Z-score (N = 928).

Characteristics	N (Weighted %)					Chi-square (*p* value)
	All	Underweight	Normal	Overweight	Obese	
N	928 (100)	59 (6.5)	488 (52.3)	193 (21.3)	182 (19.9)	
Age (years) (mean ± SE)	12.1 ± 0.1	12.2 ± 0.3	12.3 ± 0.1	12.3 ± 0.2	11.7 ± 11.3	3.0 (0.031*)
Sex						
Boy	311 (33.6)	18 (12.1)	152 (31.1)	53 (27.0)	86 (48.2)	23.1 (<0.001*)
Girl	617 (66.4)	41 (70.9)	336 (68.9)	140 (73.0)	96 (51.8)	
Ethnicity						
Malay	852 (91.9)	55 (93.8)	454 (93.1)	176 (90.9)	161 (88.8)	16.7 (0.068)
Chinese	8 (0.9)	–	7 (1.5)	–	1 (0.5)	
Indian	34 (3.9)	4 (6.2)	10 (2.3)	11 (6.1)	9 (5.4)	
Others	34 (3.3)	–	17 (3.1)	6 (3.0)	11 (5.3)	
School category						
Primary	465 (51.7)	31 (49.8)	229 (48.7)	90 (47.9)	111 (64.1)	14.3 (0.004*)
Secondary	463 (48.3)	28 (50.2)	259 (51.3)	103 (52.1)	71 (35.9)	
Family structure						
Both parents	857 (92.8)	53 (89.6)	447 (91.4)	181 (93.6)	176 (96.7)	6.8 (0.094)
Single parent	65 (7.2)	6 (10.4)	41 (8.6)	12 (6.4)	6 (3.3)	
Asthma						
No	816 (88.4)	54 (90.6)	429 (87.6)	178 (92.4)	155 (85.3)	5.5 (0.175)
Yes	106 (11.6)	5 (9.4)	59 (12.4)	15 (7.6)	27 (14.7)	
Bullied						
No	763 (84.4)	45 (78.4)	406 (84.8)	175 (90.7)	137 (78.4)	12.4 (0.009*)
Yes	143 (15.6)	13 (21.6)	74 (15.2)	18 (9.3)	38 (21.6)	
Mother’s highest education						
No formal education	13 (1.6)	3 (5.6)	4 (0.9)	3 (1.7)	3 (1.9)	7.5 (0.366)
Low education	607 (66.3)	38 (64.6)	321 (66.3)	128 (66.5)	120 (66.7)	
High education	287 (32.1)	17 (29.8)	152 (32.7)	60 (31.8)	58 (31.4)	
Mother’s highest education						
No formal education	13 (1.6)	3 (5.6)	4 (0.9)	3 (1.7)	3 (1.9)	7.5 (0.366)
Low education	607 (66.3)	38 (64.6)	321 (66.3)	128 (66.5)	120 (66.7)	
High education	287 (32.1)	17 (29.8)	152 (32.7)	60 (31.8)	58 (31.4)	
Father’s highest education						
No formal education	14 (1.8)	3 (4.5)	7 (1.9)	1 (0.5)	3 (1.9)	5.4 (0.520)
Low education	585 (66.4)	34 (59.2)	304 (66.9)	128 (69.1)	119 (66.7)	
High education	271 (31.9)	19 (36.4)	140 (32.1)	57 (30.5)	55 (31.4)	
Household monthly income						
Low income	245 (28.4)	16 (29.5)	140 (30.4)	45 (25.5)	44 (26.0)	12.3 (0.078)
Middle income	335 (37.9)	19 (34.1)	159 (33.9)	87 (47.9)	70 (38.8)	
High income	297 (33.7)	20 (36.4)	163 (35.7)	51 (26.6)	63 (35.2)	
Self-esteem (mean ± SE)	38.4 ± 0.2	38.4 ± 0.6	38.6 ± 0.3	38.2 ± 0.5	38.3 ± 0.5	14.0 (0.599)

**Data not available fo**r: 4 in tuition attendance; 16 in bullied; 41 in school absenteeism; 15 in mother’s education status; 52 in father’s education status; 45 in monthly household income; 4 in self-esteem, 6 in weight status.

* Significant at *p *< 0.05.

### Comparison of HRQOL across weight status

Table 2 presents the comparison of PedsQL scores between children with normal weight, overweight, and obesity, controlling for age, sex, ethnicity, and household income. The general linear model showed significant differences in total [F(815) = 7.1, *p* = 0.001], physical functioning [F(816) = 14.5, *p* < 0.001], social functioning [F(816) = 6.3, *p* = 0.002], and psychosocial health summary [F(815) = 3.7, *p* = 0.025] scores across the different weight status categories (Table 2). The post-hoc Bonferroni test revealed that children with obesity reported significantly lower scores compared to those with normal weight [t(816) = 3.7, *p* < 0.001] and overweight [t(816) = 3.7, *p* < 0.001], but not between children with normal weight and overweight (Result not shown). There is no statistically significant difference in emotional and school functioning (Table 2).

**Table 2.** Comparison of PedsQL Scores Between Weight Status (N = 863).

Weight status	Mean ± SE					
	Physical functioning	Emotional functioning	Social functioning	School functioning	Psychosocial health summary	Total score
Normal	82.9 ± 1.5	72.7 ± 1.6	83.4 ± 1.6	72.0 ± 1.5	76.0 ± 1.3	77.8 ± 1.3
Overweight	81.3 ± 2.0	72.1 ± 2.0	82.1 ± 1.8	73.0 ± 1.8	75.9 ± 1.5	77.3 ± 1.4
Obese	74.8 ± 1.7	70.0 ± 2.0	77.1 ± 2.0	69.6 ± 1.6	72.2 ± 1.5	72.8 ± 1.4
F	14.5	1.1	6.3	1.8	3.7	7.1
*p* value	<0.001**	0.346	0.002**	0.173	0.025*	0.001**

Controlling for age, ethnicity, sex, and household income.

*p < 0.05.

**p < 0.01.

### Factors associated with HRQOL

In the final model for total PedsQL, children with a higher BMI z-score (obesity), a history of being bullied, and from single-parent families were shown to have significantly lower scores [*β*_obesity _= −3.39, *t*(363) = −2.49, *p *= 0.015; *β*_bullied _= −7.14, *t*(363) = −3.16, *p *= 0.002; *β*_single parent _= −7.34, *t*(363) = −2.12, *p *= 0.035], while those with higher self-esteem reported significantly higher scores [*β *= 0.94, *t*(363) = −8.28, *p *< 0.001]. Meanwhile, only the degree of overweight and self-esteem scores were shown to be statistically significant predictors for the physical functioning domain (*p *< 0.05). Children with obesity reported significantly lower physical functioning scores than children who were overweight [*β *= −4.50, *t*(363) = −2.49, *p *= 0.014]. Whereas, for every score increase in RSES, the physical functioning score is expected to increase by 0.822 [*β *= 0.82, *t*(363) = 5.70, *p *< 0.001] (Table 3).

**Table 3.** Predictors of HRQOL Among Overweight and Obese Children (N = 375).

Predictors	Total PedsQL		Physical Functioning		Psychosocial Health Summary	
	Coefficient	t value (*p* value)	Coefficient	t value (*p* value)	Coefficient	t value (*p* value)
Girl (boy as reference)	−0.839	−0.58 (0.563)	−1.767	−0.94 (0.346)	−0.452	−0.29 (0.774)
Age (continuous variable)	0.422	0.75 (0.456)	0.276	0.74 (0.459)	0.444	0.71 (0.477)
Malay (non-Malay as reference)	–	–	7.289	1.93 (0.055)	–	–
Secondary school (primary school as reference)	−3.499	−1.30 (0.194)	–	–	−4.224	−1.40 (0.163)
Obese (overweight as reference)	−3.392	−2.49 (0.015*)	−4.497	−2.47 (0.014*)	−2.777	−1.81 (0.071)
Bullied (no as reference)	−7.138	−3.16 (0.002**)	−5.591	−1.84 (0.067)	−7.624	−3.29 (0.001**)
Mother’s education (ordered categorical variable)	1.073	0.65 (0.516)	2.568	1.14 (0.256)	0.060	0.04 (0.972)
Father’s education (ordered categorical variable)	2.576	1.64 (0.102)	1.689	0.80 (0.427)	2.790	1.69 (0.093)
Household income (ordered categorical variable)	−0.088	−0.05 (0.958)	–	–	0.730	0.42 (0.676)
Single parent family (both parents’ family as reference)	−7.343	−2.12 (0.035*)	−7.167	−1.70 (0.089)	−6.936	−1.88 (0.061)
Asthma (no as reference)	–	–	−4.024	−1.42 (0.155)	–	–
Self-esteem (continuous variable)	0.941	8.28 (<0.001**)	0.822	5.70 (<0.001**)	0.981	7.70 (<0.001**)

*p < 0.05.

**p < 0.01.

For the psychosocial health summary, history of being bullied and self-esteem scores were shown to be statistically significant predictors. Those with a history of being bullied reported significantly lower psychosocial health summary scores [*β *= −7.62, *t*(363) = −3.29, *p *= 0.001]. In terms of self-esteem, for every score increase in RSES, the psychosocial health summary score is expected to increase by 0.981 [*β *= 0.98, *t*(363) = 7.70, *p *< 0.001] (Table 3).

There is no significant association observed between other factors (gender, age, ethnicity, school category, presence of asthma, parents’ educational status, and household income) and HRQOL (total score, physical functioning, and psychosocial health summary). The history of being bullied was not significantly associated with physical functioning, while single parenting was not a significant predictor of psychosocial health summary (Table 3).

## Discussion

A significant association was found between HRQOL and weight status, as children with obesity reported significantly lower scores than children who were normal weight and overweight (6.4% lower in both cases) (Table 2). The significant inverse relationship between weight status and HRQOL was also reported in previous studies (Al-Akour et al., 2012; Anderson et al., 2017; Brodsgaard et al., 2016; Meixner et al., 2020). As HRQOL is multidimensional, some domains may be more affected by obesity status compared to others. In this study, children with obesity had significantly lower HRQOL in the domains of physical functioning (9.8% and 7.8% lower), social functioning (7.6% lower in both cases), and physical health summary (5.0% and 4.9% lower) when compared to children who were normal weight and overweight, respectively (Table 2).

Children with obesity are more likely to experience poor body image and self-esteem, with higher severity than adult-onset obesity, since mid-childhood is the crucial period for self-esteem development and body image (Lee, 2009). There is a growing number of literature showing that children with obesity were targets of social stigmatisation and weight-based teasing (Bacchini et al., 2015; Ibrahim et al., 2019; Puhl et al., 2017; Wong et al., 2019). A study among children aged 8–12 years found that children who were overweight reported significantly higher self-stigma and lower HRQOL compared to peers with normal weight, with a strong negative correlation between self-stigma and HRQOL among those who were overweight (Wong et al., 2019). Internalisation of this weight stigma in youths with overweight and obesity was found to be associated with reduced physical activity, lower self-esteem, and increased depression, anxiety, suicidality, and body dissatisfaction (Puhl & Latner, 2007). A 15-year longitudinal study suggested that experiencing weight teasing during adolescence may result in maladaptive eating behaviours, body dissatisfaction, and obesity in adulthood (Puhl et al., 2017).

In the Malaysian context, weight-based teasing has been found to be significantly associated with overweight and obesity among Malaysian adolescents (Ibrahim et al., 2019). However, longitudinal studies are needed to confirm this finding due to the limitations of the study design. To our knowledge, Malaysia is still lacking studies that focus on exploring social contexts such as the stigmatization of childhood obesity. In fact, the social impact of obesity has been included as one of the scopes in the latest Nutrition Research Priorities in Malaysia For 12th Malaysia Plan (2021–2025) (National Coordinating Committee on Food and Nutrition, 2020).

In the final model, lower self-esteem was found to be a significant predictor of lower total HRQOL, physical and psychosocial functioning, in agreement with the findings of other studies (Kim et al., 2013; Kolodziejczyk et al., 2015). Stern et al. (Stern et al., 2007) reported self-esteem as a partial mediator of the relationship between weight-related teasing and QOL among adolescents with obesity. As obesity often leads to stigmatisation and discrimination that results in social marginalisation, it may have a negative impact on HRQOL. Children with overweight and obesity are susceptible to weight stigma and bias from various sources. Although it is common that peers often show negative attitudes towards youth with obesity, there are growing studies reporting that parents and educators are also showing weight stigma towards children (Berge et al., 2016; Pont et al., 2017; Puhl et al., 2013).

In the total HRQOL and psychosocial functioning model, children with overweight and obesity who reported a history of being bullied had a significantly lower score in HRQOL compared with those who had never been bullied (Table 3). This finding is consistent with reports from other studies (Gunawardana et al., 2021; Haraldstad et al., 2011; Radhakishun et al., 2016; Stern et al., 2007). Bullying is defined as systematic abuse of power or repetitive, intentional aggressive behaviour towards a victim who cannot defend himself or herself (Smith, 2004). Children who are bullied or victimised may be marginalised from the peer group. As a result, their access to social interaction with peers, which offers a role model for more fitting social skills and protection from further bullying, is limited (Nansel et al., 2004). Children with obesity who are severely upset because of peer bullying may internalise the content of the assault, which contributes to negative self-acknowledgement and elevates depressive symptoms (Storch et al., 2005).

Looking at the degree of overweight, children with obesity showed poorer HRQOL than children who were overweight in total score and physical functioning. Al-Akour et al. (Al-Akour et al., 2012) stated in their study that adolescents with obesity reported significantly lower scores than children who were overweight, which is in agreement with another study (Wake et al., 2010). Having obesity might make it more difficult for children to run and take part in sports and make them more likely to have less energy, thus deteriorating their physical functioning. Tsiros et al. (Tsiros et al., 2016) examined the relationship between obesity and physical functioning among 10 – to 13-year-old children and found that children with a higher body fat percentage showed lower physical activity and physical functioning. They suggested reducing body fat percentage as the goal target of intervention to improve physical functioning among children with obesity (Tsiros et al., 2016).

Children with overweight and obesity who were from single-parent families reported significantly lower HRQOL scores in the final model than those who lived with both parents, which confirms the findings from other studies (Simon et al., 2008; Swallen et al., 2005; Williams et al., 2005). In definition, a single-parent family (comprising a single parent with one or more children) occurs either after divorce or resulting from the death of a spouse or partner (Sănduleasa et al., 2011). A single-parent family has been found to inflict a negative effect on the cognitive, emotional, and social development of children (Mabuza & Okeke, 2004; Uchenna, 2013), thus affecting their QOL. Despite being more vulnerable to financial constraints, single parents also face difficulty exercising their parental role, especially in developing a good parent–child relationship (Zeiders et al., 2011), which is a crucial element to building a child's self-esteem (Krauss et al., 2020). Moreover, studies showed that parental involvement in the obesity prevention programme, combined with nutrition and exercise intervention for children, was more effective for both parents and children compared to intervention for children alone (Kim et al., 2016; Lee et al., 2017). Additionally, gender was not a significant predictor of HRQOL in the final model, which is consistent with findings from other studies (Haraldstad et al., 2011; Hughes et al., 2007; Schwimmer et al., 2003; Stern et al., 2007).

### Study strengths and limitations

Our study has several strengths. First, this was the only study that explored the factors associated with HRQOL among children with overweight and obesity in Malaysia, thus providing valuable new knowledge in childhood obesity research. Other than that, we analysed the data using the complex sample analysis procedure that aligned with the sampling design. The complex sample analysis took into account the sample weight, which specified the different possibilities of selection of the individuals in a complex sample.

The limitation of this study would be the cross-sectional design, which makes it difficult to determine the direction of the association. In addition, generalising the findings to other ethnicities could also be a limitation, as 91.9% (n = 852) of the participants were Malays while only 8.1% (n = 76) of them were non-Malays. Furthermore, since this study was conducted among schoolchildren in Kuala Lumpur, there is also the issue of generalizability of the findings to schoolchildren from other states.

Lastly, only a general question about the history of bullying was included in the questionnaire, which limited the interpretation of how this factor affected HRQOL. Children may be teased on matters such as their appearance, weight, behaviour, family, gender, faith, health/medical issues, abilities, clothing, and intelligence. Future research should focus on investigating the types and sources of bullying. Moreover, the perspectives of parents and teachers on childhood obesity need to be explored so that predictors of potential misconceptions and barriers could be identified and intervened. Addressing childhood obesity requires a comprehensive approach that warrants active involvement from multiple parties, especially parents and teachers.

### Public health implications

Obesity prevention programmes and interventions should be introduced during early childhood and adolescence. The principle of intervention needs to cover not only physical activity and healthy eating components but also the psychosocial aspect as well. This study also conveys the message that more attention and focus need to be channelled on secondary prevention in the management of psychosocial impacts of childhood obesity. The undetected psychosocial issues affecting children with overweight and obesity in the population might be a silent killer of their mental health, which further compromises their adulthood. In Malaysia, mental health screening using the Depression, Anxiety, and Stress Scale (DASS) has been implemented among Form Four (16-year-old) secondary school students through the Healthy Mind Programme. Although this routine screening is essential for early detection of psychological problems, more comprehensive and targeted screenings for high-risk populations, such as children with obesity are needed. Given policy changes, there should be a definite guideline on the referral of abnormal weight status to health clinics, and the management of obesity among children need to be holistic as it requires active involvement from family and schools. Screening of psychological problems among children with overweight and obesity and adolescents needs to become a routine beyond obesity treatment at the clinic but also by counsellors at school.

## Conclusion

Children with obesity were reported to have significantly poorer HRQOL compared to children who were normal weight and overweight. Among children with overweight and obesity, those with obesity, had a history of being bullied, reported low self-esteem, and lived with a single parent were found to have significantly lower HRQOL. This latest finding should alarm not only parents and schools but also policymakers about the importance of incorporating those factors into interventions and obesity prevention programmes, either school – or community-based.
